# A public health perspective to environmental barriers and accessibility problems for senior citizens living in ordinary housing

**DOI:** 10.1186/s12889-016-3369-2

**Published:** 2016-08-11

**Authors:** Marianne Granbom, Susanne Iwarsson, Marianne Kylberg, Cecilia Pettersson, Björn Slaug

**Affiliations:** Department of Health Sciences, Lund University, Box 157, 221 00 Lund, Sweden

**Keywords:** Ageing population, Built environment, Functional capacity, Health, Policy, Ageing-in-place

## Abstract

**Background:**

Housing environments that hinder performance of daily activities and impede participation in social life have negative health consequences particularly for the older segment of the population. From a public health perspective accessible housing that supports active and healthy ageing is therefore crucial. The objective of the present study was to make an inventory of environmental barriers and investigate accessibility problems in the ordinary housing stock in Sweden as related to the functional capacity of senior citizens. Particular attention was paid to differences between housing types and building periods and to identify environmental barriers generating the most accessibility problems for sub-groups of senior citizens.

**Methods:**

Data on environmental barriers in dwellings from three databases on housing and health in old age was analysed (*N* = 1021). Four functional profiles representing large groups of senior citizens were used in analyses of the magnitude and severity of potential accessibility problems. Differences in terms of type of housing and building period were examined.

**Results:**

High proportions of one-family houses as well as multi-dwellings had substantial numbers of environmental barriers, with significantly lower numbers in later building periods. Accessibility problems occurred already for senior citizens with few functional limitations, but more profound for those dependent on mobility devices. The most problematic housing sections were entrances in one-family houses and kitchens of multi-dwellings.

**Conclusions:**

Despite a high housing standard in the Swedish ordinary housing stock the results show substantial accessibility problems for senior citizens with functional limitations. To make housing accessible large-scale and systematic efforts are required.

## Background

The demographic development is making housing provision for senior citizens a burning issue, and there is a growing interest on ageing and the role of the built environment to foster active and healthy ageing [1, 2]. The home environment is the primary context for senior citizens to perform daily activities and to participate in social life. Moreover, accessible and adequately designed dwellings support continued independence at old age, which is associated with positive health aspects [3, 4]. The conditions and characteristics of the built environment in the current housing stock are therefore important to examine from a public health perspective.

In many countries different housing options for senior citizens are developing and transforming in both content and design. Due to a strong ageing-in-place policy in many Western countries and in Sweden in particular, [4] an increasing share of the ageing population can be expected to live in dwellings in the ordinary housing stock despite health problems and need of health- and social care services that often come with age. In Sweden, 1.8 million people aged 65 or older (95 %) live in ordinary housing; approximately half live in multi-dwelling blocks and half live in one-family houses [5]. Out of the total ordinary housing stock in Sweden (4.5 million dwellings, Dec 31, 2013) 28 % are occupied by at least one individual aged 65+ [5]. This situation puts a high demand on society to provide ordinary housing that meets the needs of senior citizens also if health problems and functional limitations gradually emerge. As yet, the body of research that has addressed such topics is limited.

In general, the housing standard in Sweden is high [6], but for the ordinary housing stock to be suitable to all senior citizens regardless of functional capacity implies specific demands on accessibility. It is estimated that half of all senior citizens living in multi-dwellings live in buildings lacking lifts, and for many of those having lifts, steps or other level differences still need to be forced in order to enter or exit the dwelling. Furthermore, it is estimated that a similar proportion (49 %) lives in dwellings with a bathtub instead of shower place, which constitutes a fall risk [7]. Since at least 25 years, building legislation and housing standards in Sweden have incorporated aspects on accessibility for all citizens [8, 9]. However, since the production of new dwellings is very low the effects of such directives do not keep pace with the rapid demographic change.

Even though life expectancy has increased globally as well as in Sweden, the number of years lived with disability has not decreased [10]. That is, multi-morbidity increases markedly with age and with that also functional limitations and disabilities [11, 12]. The complexity of combinations of functional limitations and the functional trajectories that are characteristic for the ageing process are seldom accounted for in research but is important for public health policies to consider [1, 13].

Accessibility can be defined as the interaction between the demands of the physical environment (E) and the individual’s functional capacity (P). Accessibility is supported theoretically by the Ecological Model of Ageing [14] and is one aspect of Person-Environment (P-E) fit. According to Lawton & Nahemow [14], adaptive behaviour is the outcome of P-E fit. Thus, accessibility is influential on disability, and this kind of theorizing is well in line with more recent bio-psychosocial models such as the International Classification of Functioning, Disability and Health (ICF) [15] that emphasise the importance of environmental factors for disability. Accessibility is usually considered as an objective aspect of housing; the E component is described on the basis of national guidelines and standards for design and the P component is described based on professional assessments [16].

Housing accessibility problems are commonly dealt with on an individual level providing housing adaptations. However, aiming for accessible housing that supports ageing-in place, measures must be taken also on a group or population level. Foresighted housing provision with potential to accommodate the ageing population requires knowledge on the occurrence of environmental barriers in the ordinary housing stock. However, valid information on the detailed level required is lacking and the consequences environmental barriers generate in terms of accessibility problems for those senior citizens having different combinations of functional limitations are unknown.

The objective of the present study was to make an inventory of environmental barriers and investigate accessibility problems in the ordinary housing stock in Sweden as related to the functional capacity of senior citizens. Particular attention was paid to differences between types of housing and building periods. A specific aim was to identify the environmental barriers that generate the most accessibility problems for sub-groups of the ageing population with different combinations of functional limitations.

## Methods

### Study context

The present study made use of data on the physical home environment in ordinary dwellings from three separate research projects on housing and health, all based on data collected with older people in the south of Sweden. The ENABLE-AGE Project (EA Project) targeted very old people, living alone in ordinary housing in five European countries; for the present study data from the Swedish sub-sample were used (*N* = 397; mean age = 85 years; 80–89 years old). The participants were randomly selected from National Public Register [17] based on the inclusion criteria living alone in ordinary housing, stratifying for 25 % men. The study district contained three strategically selected municipalities with mainly urban and semi-urban areas (Helsingborg, Halmstad and Lund). The Home and Health in People Ageing with Parkinson’s Disease Project (PD Project) targeted people with Parkinson’s disease (PD). Participants diagnosed with PD more than one year ago were recruited via Neurology departments at three Skåne County hospitals (*N* = 255, mean age = 70 years, range 45–93 years) [18]. The study area was limited to the catchment area of the hospitals i.e., several municipalities in Skåne County containing both urban, semi-rural and rural areas. The Home and Health in the Third Age Project (Third Age Project) targeted younger senior citizens and included 371 participants living in ordinary housing in Skåne County (mean age = 68 years, range 67–70 years) [19]. The sample of The Third Age project was made by random sampling of all inhabitants at the specific age group in five municipalities in Skåne County (Eslöv, Osby, Malmö, Hässleholm and Ystad). This project was a part of the Swedish National Study on Ageing and Care (SNAC), targeting age groups ranging from 66 to 81 years [20]. For all three projects data were collected at home-visits by experienced data collectors that underwent project-specific training. For details, see [17–19].

### Sample of dwellings

Due to internal missing data for two observations, the pooled sample (*N* = 1021) comprised 662 dwellings in multi-dwelling blocks (65 %) and 359 one-family houses (35 %). Different kinds of tenure were represented. The dwellings were situated in 34 municipalities in the south of Sweden (ranging from 7,500 to 320,000 inhabitants) representing urban, semi-rural and rural districts. For dwellings where the year of build was known (*n* = 609; not available for the Third Age Project), 39 % were built before 1960, (*n* = 236), 37 % were built 1960–1979 (a period in time in Sweden with massive multi-dwelling block construction; *n* = 225) and 24 % were built 1980 or later (a period dominated by one-family houses construction; *n* = 148).

### Measures

Descriptive data on type of housing and tenure as well as year of build in the EA Project were collected with project-specific questions. For the PD Project data on year of build were retrieved from the Swedish National Board of Housing, Building and Planning. For the Third Age Project such information was not possible to retrieve.

**Fig. 1:**
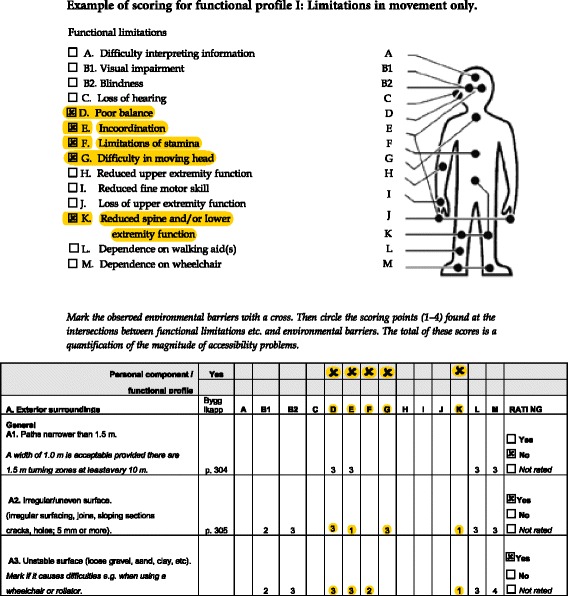
Example of the generation of accessibility problem score for functional profile 1 by combining functional limitations of the profile with environmental barriers present in a specific dwelling

Environmental barriers were assessed by means of the environmental (E) component of the version of the Housing Enabler Instrument (HE) available at the time for data collection. The environmental component of HE includes professional observation by trained data collectors of the presence (yes) /absence (no) of environmental barriers, defined according to national standards and guidelines for housing design. For the present study we used a reduced list of 60 environmental barriers (27 indoors, 13 at entrances and 20 in the close exterior surroundings) representing the core barriers in terms of detecting accessibility problems [21]. The HE also includes a personal (P) component for assessments (interview and observation) of presence (yes) /absence (no) of functional limitations and dependence on mobility devices (14 items, displayed in Fig. 1). The magnitude of accessibility problems in a case is calculated by combining the E and P components using a scoring matrix (see Fig. 1). For each intersection between the two components, the matrix assigns predefined severity ratings (0–4) that are summed up to an accessibility problem score, which represents a quantification of predicted problems (theoretical range for the reduced list, 0–904). For individuals with no functional limitations or dependence on mobility devices, this score is always 0; higher scores indicate greater accessibility problems. The HE is an internationally acknowledged, reliable and valid instrument available in several languages [22]. The validity of the HE has been successively optimised during 20 years of research, including empirical studies of more than 2000 senior citizens and their dwellings across Europe [14]. Sufficient inter-rater reliability has been demonstrated in several studies, in Sweden and other countries [23–26]. The reduced list used in the present study was obtained through a rigorous research process, utilizing statistically defined criteria and an expert panel approach. The validity of the reduced version was demonstrated by close to perfect rank correlations between the accessibility scores generated by the original and reduced versions [18].

### Data analyses

In order to arrive at more generalizable results, we did not use any person-related data of the individuals actually living in the dwellings. Instead, to analyse accessibility problems at group level we used functional profiles developed through simulations and statistical analyses of large data sets on functional limitations of older people from previous studies with the Housing Enabler [27]. The functional profiles were based on a categorization of the 14 functional limitations into broader categories (see Table 5 in the Appendix). Four functional profiles with increasing complexity were used; Profile I: limitations in movement only; Profile II: limitations in movement and upper extremity; Profile III: limitations in movement, upper extremity and dependence on mobility devices; and Profile IV: limitations in movement, upper extremity, dependence on mobility devices and loss of sight. To explore and estimate the accessibility problem score for all profiles in all dwellings, each functional profile was combined with the environmental barriers present in each dwelling, using the scoring matrix (Fig. 1). However, since these analyses targeted profiles with functional limitations items joined into broader categories, we applied a somewhat modified scoring procedure. We first calculated the average rating for each category of functional limitations, and then summed up these average ratings to an accessibility problem score.

Descriptive statistics were used to present environmental barriers and accessibility scores. Differences due to housing type and building period were tested by means of Kruskal-Wallis test. Differences in accessibility score due to functional profiles and building period were tested by means of Kruskal-Wallis test and also displayed graphically.

The HE Software and SAS 9.3 software were used for the analyses. The level for statistical significance was set at *p* < 0.05.

## Results

The median number of environmental barriers present was 31 (of 60) in multi-dwelling blocks and 32 in one-family houses (*p* = 0.003). Environmental barriers indoors were more frequent in one-family houses (*p* < 0.001). For details, see Table 1.

**Table 1:** Number of environmental barriers in dwellings of senior citizens in ordinary housing, according to housing type (*N* = 1021)

	Multi-dwelling block	One-family house			
	(*n* = 662)	(*n* = 359)			
	Md (q1-q3)		*p*-value	Effect size^b^	95 % CI
Exterior surrounding (0–20^a^)	9 (7–11)	7 (5–9)	<0.001	−2.0	−2.0; −1.0
Entrances (0–13^a^)	7 (5–9)	6 (5–7)	<0.001	−1.0	−1.0; −1.0
Indoors (0–27^a^)	15 (13–17)	18 (17–20)	<0.001	3.0	3.0; 4.0
In total (0–60^a^)	31 (27–35)	32 (28–35)	0.003	1.0	0.0; 2.0

Note Md (q1-q3) Median and (first quartile - third quartile), CI confidence interval

^a^Possible range

^b^Location shift, Hodges-Lehmann estimation

There were fewer environmental barriers in dwellings from later building periods than earlier (*p* < 0.001), and most notably so in exterior surroundings and entrances (Table 2). With the exception of for environmental barriers indoors, a smaller but significant difference was seen for one-family houses, with fewer environmental barriers in total for those built in later periods.

**Table 2:** Number of environmental barriers in dwellings of senior citizens in ordinary housing, according to building period (*N* = 609)

Housing type	Built before 1960	Built in 1960-1979	Built after 1979							
	Md (q1-q3)			*p*-value	“Effect size”^b^	95 % CI	“Effect size”^b^	95 % CI	“Effect size”^b^	95 % CI
Multi-dwelling block (*n* = 416)	(*n* = 141)	(*n* = 153)	(*n* = 122)		Before 1960 vs. 1960-1979		Before 1960 vs. after 1979		1960-1979 vs. after 1979	
Exterior surrounding (0–20^a^)	10 (8–11)	9 (7–11)	8 (6–9)	<0.001	0.0	−1.0; 0.0	−2.0	−2.0; −1.0	−1.0	−2.0; −1.0
Entrances (0–13^a^)	8 (7–9)	6 (5–8)	5 (4–6)	<0.001	−2.0	−2.0; −1.0	−3.0	−4.0; −3.0	−1.0	−2.0; −1.0
Indoors (0–27^a^)	14 (12–17)	14 (12–15)	13 (11–15)	0.002	−1.0	−1.0; 0.0	−1.0	−2.0; −1.0	−1.0	−1.0; 0.0
Total (0-60^a^)	32 (29–35)	30 (26–33)	26 (22–29)	<0.001	−3.0	−4.0; −2.0	−6.0	−7.0; −5.0	−4.0	−5.0; −2.0
One-family house (*n* = 193)	(*n* = 95)	(*n* = 72)	(*n* = 26)		Before 1960 vs. 1960-1979		Before 1960 vs. after 1979		1960-1979 vs. after 1979	
Exterior surrounding (0–20^a^)	8 (7–10)	7 (5–9)	7 (6–9)	0.004	−1.0	−2.0; 0.0	−1.0	−2.0; 0.0	0.0	−1.0; 1.0
Entrances (0–13^a^)	7 (6–8)	6 (5–8)	5 (3–6)	0.005	0.0	−1.0; 0.0	−2.0	−3.0; −1.0	−1.0	−2.0; 0.0
Indoors (0–27^a^)	18 (16–21)	18 (17–20)	18 (16–19)	0.178	0.0	−1.0; 0.0	−1.0	−3.0; 0.0	−1.0	−2.0; 0.0
Total (0–60^a^)	33 (30–36)	31 (28–36)	30 (26–32)	0.001	−2.0	−4.0; 0.0	−4.0	−6.0; −2.0	−2.0	−4.0; 1.0

Note Md (q1-q3) Median and (first quartile - third quartile) CI confidence interval

^a^Possible range

^b^Location shift, Hodges-Lehmann estimation

**Fig. 2:**
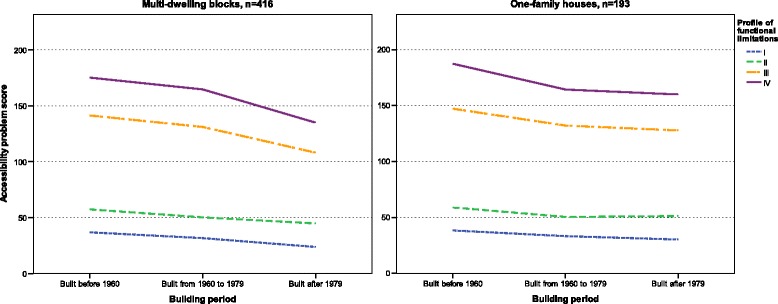
Accessibility problems for senior citizens in multi-dwelling blocks and one-family houses from different building periods based on four different functional profiles (I: functional profile with limited mobility; II: functional profile with limited mobility and limited upper extremity function; III: functional profile with limited mobility, limited upper extremity function and dependence on mobility devices and; IV: functional profile with limited mobility, limited upper extremity function, dependence on mobility devices and visual impairment)

Regardless of housing type and building period, all dwellings would give accessibility problems for older people with any of the four functional profiles. Statistical testing showed highly significant differences between all four profiles (*p* < 0.0009), regardless of building period. Dwellings built before 1960 generated more accessibility problems than newer dwellings (Fig. 2). Differences in accessibility problems between housing types were small, ranging from no difference to 35 accessibility problem score. With a more complex functional profile the higher the accessibility problem scores in all dwellings. For multi-dwellings built before 1960 profile IV generated an accessibility problem score 3.7 times higher compared to profile I.

Indoors in multi-dwellings, among the top-ten environmental barriers causing accessibility problems, several were found in kitchens. For one-family houses the barriers were more evenly distributed on the sections kitchens, bathrooms and indoors in general. Three barriers were among top-ten barriers regardless of housing type or functional profile; *steps/threshold/differences in level between rooms; wall-mounted cupboards* and *shelves placed too high in kitchen and no grab bars at shower/bath and toilet* (Table 3). One-family houses had more top-ten environmental barriers causing accessibility problems at entrances than in exterior surroundings, in multi-dwellings they were more evenly distributed. Regardless of housing type or functional profile; *irregular/uneven surface in exterior surroundings*; *high thresholds and/or steps at the entrance*; *stairs the only route (no lift/ramp)* and *storage areas can only be reached* via *steps or other difference in level* were among the top-ten barriers (Table 4).

**Table 3:** Rank order of the environmental barriers contributing most to the accessibility problem score indoors, according to functional profile and housing type (*N* = 1021)

Environmental barrier (*n* = 27)	Multi-dwelling block (*n* = 662)				One-family house (*n* = 359)			
	Profile I.^a^	Profile II.^b^	Profile III.^c^	Profile IV.^d^	Profile I.^a^	Profile II.^b^	Profile III.^c^	Profile IV.^d^
Indoors in general								
Steps/threshold/differences in level between rooms	**3**	**10**	**5**	**2**	**3**	**10**	**5**	**3**
Narrow passages/corridors	^e^	^e^	17	17	^e^	^e^	15	18
Narrow doors	^e^	^e^	12	13	^e^	^e^	**8**	**9**
Complex manoeuvres required	14	12	21	20	20	18	26	25
Use require hands	^e^	**3**	**10**	11	^e^	**5**	13	16
Controls in high/inaccessible position	12	**7**	**4**	**6**	12	**7**	**3**	**4**
Stairs to upper storey with necessary dwelling functions	19	22	24	24	**6**	13	17	**10**
Stairs to basement with necessary dwelling functions	22	25	27	27	**8**	15	21	12
No handrails/handrail only on one side	20	23	25	25	**4**	12	19	17
Handrails too short	21	24	26	26	**5**	14	22	19
Kitchen								
Insufficient manoeuvring spaces around white goods	**5**	**9**	14	**5**	**7**	11	14	**7**
Wall-mounted cupboards and shelves placed too high	**1**	**1**	**1**	**1**	**2**	**1**	**2**	**1**
No surface at a height suitable for sitting while working	**9**	18	**8**	**9**	16	22	12	15
Shelves too deep	**8**	**5**	**3**	**4**	15	**6**	**7**	**8**
Complex manoeuvres required	15	13	22	21	18	16	24	23
Use require hands	^e^	**2**	**9**	**10**	^e^	**3**	**10**	13
Controls in high/inaccessible position	13	**8**	**7**	**8**	13	**8**	**4**	**5**
Bathroom								
Insufficient manoeuvring spaces where turning is necessary	11	20	**6**	**7**	17	23	**9**	11
Insufficient space for stool/bath board in shower/bath	**10**	19	19	19	22	25	27	27
No grab bars at shower/bath and toilet	**2**	**6**	**2**	**3**	**1**	**2**	**1**	**2**
Complex manoeuvres required	18	16	23	23	19	17	25	24
Use require hands	^e^	**4**	11	12	^e^	**4**	11	14
Controls in high/inaccessible position	17	11	13	14	14	**9**	**6**	**6**
Wash-basin placed at height for use only when standing	**4**	14	16	16	**9**	19	18	21
Toilet 47 cm or lower	**7**	17	18	18	**10**	20	16	20
Shower stall with kerb/level difference	16	21	20	22	11	21	20	22
Bathtub instead of shower	**6**	15	15	15	21	24	23	26

Note: Top-10 barriers of each functional profile and housing type are bolded

^a^Profile I: Functional profile with limited mobility

^b^Profile II: Functional profile with limited mobility and limited upper extremity function

^c^Profile III: Functional profile with limited mobility, limited upper extremity function and dependence on mobility devices

^d^Profile IV: Functional profile with limited mobility, limited upper extremity function, dependence on mobility devices and loss of sight

^e^Did not generate any accessibility problem score

**Table 4:** Rank order of the environmental barriers contributing most to the accessibility problem score in the close exterior surroundings and at entrances, according to functional profile and housing type (*N* = 1021)

Environmental barrier (*n* = 33)	Multi-dwelling block (*n* = 662)				One-family house (*n* = 359)			
	Profile I.^a^	Profile II.^b^	Profile III.^c^	Profile IV.^d^	Profile I.^a^	Profile II.^b^	Profile III.^c^	Profile IV.^d^
Exterior surroundings								
Paths narrower than 1.5 m	24	25	26	30	12	15	18	18
Irregular/uneven surface	**7**	**10**	**8**	**5**	**5**	**7**	**5**	**5**
Unstable surface	18	20	19	20	**8**	12	**8**	**8**
Steep gradient	19	21	24	22	20	21	23	23
Routes with steps	27	27	31	32	14	16	21	21
No/insufficient tactile cues of abrupt level change	^e^	^e^	^e^	28	^e^	^e^	29	29
High kerbs	**2**	**5**	**7**	**10**	**9**	13	17	17
Kerb ramps with steep gradients	15	17	18	24	19	20	28	28
No handrails on steep gradients	16	18	30	31	16	17	30	30
No resting surface or too long distances between them on slopes	21	22	29	33	26	27	32	32
Poor lightning along circulation paths	28	28	22	23	18	19	12	12
Passenger loading zone far from entrance	22	23	28	21	22	23	19	19
No/too few seating places	**3**	**6**	15	16	28	28	33	33
Refuse room/bin can only be reached via steps or other difference in level	**9**	12	**10**	**7**	17	18	16	16
Letterbox can only be reached via steps or other difference in level	25	26	27	25	23	24	22	22
Refuse bin difficult to reach	14	**3**	**4**	11	13	**3**	**10**	**10**
Letterbox difficult to reach	13	**2**	**2**	**8**	11	**2**	**7**	**7**
Narrow door openings	^e^	^e^	25	29	^e^	^e^	26	26
Entrances								
High thresholds and/or steps at the entrance	**6**	**9**	**6**	**4**	**2**	**4**	**2**	**2**
Insufficient manoeuvring space at doors, outside and inside	^e^	^e^	14	15	^e^	^e^	**9**	**9**
No resting area in front of entrance door	^e^	^e^	23	26	^e^	^e^	27	27
Heavy doors without automatic door opener	11	**4**	11	12	15	**8**	20	20
Doors do not stay in open position/close quickly	**10**	13	12	**9**	21	22	24	24
Complicated/illogical opening procedure	20	16	20	18	27	26	31	31
Stairs the only route (no lift/ramp)	**1**	**1**	**1**	**1**	**1**	**1**	**1**	**1**
High, low and/or irregular heights of risers	23	24	32	27	**10**	14	15	15
No handrails/handrail only on one side	12	14	17	17	**7**	11	13	14
Handrails too short	17	19	21	**3**	24	25	25	**4**
Narrow doors to sitting-out place/balcony	^e^	^e^	16	14	^e^	^e^	14	13
High threshold/level difference/step to sitting-out place/balcony	**5**	**8**	**5**	19	**4**	**6**	**4**	25
Storage areas can only be reached via steps or other difference in level	**4**	**7**	**3**	**2**	**3**	**5**	**3**	**3**
Laundry room can only be reached via steps or other difference in level	**8**	11	**9**	**6**	**6**	**10**	**6**	**6**
Inappropriate design of door to laundry room	26	15	13	13	25	**9**	11	11

Note: Top-10 barriers of each functional profile and housing type are bolded

^a^Profile I: Functional profile with limited mobility

^b^Profile II: Functional profile with limited mobility and limited upper extremity function

^c^Profile III: Functional profile with limited mobility, limited upper extremity function and dependence on mobility devices

^d^Profile IV: Functional profile with limited mobility, limited upper extremity function, dependence on mobility devices and loss of sight

^e^Did not generate any accessibility problem score

## Discussion

Despite a high housing standard in Sweden and efforts from policy-makers to improve legislation and building regulations, the results show that many environmental barriers are present in multi-dwellings as well as in one-family houses. While there is a tendency towards fewer environmental barriers in dwellings built during later periods the progress is slow and the differences between periods are small. The analyses using functional profiles show that all dwellings with environmental barriers will cause accessibility problems already for senior citizens with few functional limitations. Regardless of housing type and building period, accessibility problems get more profound for more complex functional profiles - most notably so when residents are dependent on mobility devices. It should be noted that in terms of accessibility, the problems differ between housing sections, with the most problematic environmental barriers at entrances in one-family houses and in kitchens of multi-dwellings.

Considering the increasing attention to accessibility in Swedish building legislation and housing standard over the years [8, 9], the reduction of environmental barriers over time is small. As often reflected in the public debate, it is remarkable that also newer dwellings have considerable environmental barriers. The fact that that even the most recently built multi-dwellings had as many as 26 environmental barriers (from a 60 item checklist) will lead to substantial accessibility problems for the increasing population of senior citizens, and thereby also to more health issues [4]. Moreover, the life time of a dwelling implies that the probability that at least one resident with disability will live there is as high as 55-74 % [28, 29]. This highlights the importance for the housing construction sector as well as for policy-makers to take resolute actions to avoid continuing to build dwellings with environmental barriers. It should also be noted that senior citizens more often live in older dwellings [7], where accessibility problems tend to be more extensive and more severe. Moreover, the finding that one-family houses have more environmental barriers than multi-dwellings, point to the fact that there is also an urgent need for increased knowledge on accessibility issues for all actors of the private building industry sector as well as for the general population. Efficient upgrading of the existing ordinary housing stock based on the best possible knowledge is called for [7].

The results showing that the accessibility problems will be 3–4 times higher for senior citizens having functional profile IV, than for those with profile I are striking and call for attention. Considering that reluctance to move increases with age [30] as well as the prevalence and complexity of functional limitations and disabilities over time [11, 12], the demands on barrier free housing are high. For countries like Sweden, actively supporting ageing-in-place, improved accessibility in the entire ordinary housing stock is therefore of utmost importance. Taking an international perspective, earlier research from Germany, Latvia and the USA indicates that a high prevalence of environmental barriers and accessibility problems exists in ordinary housing [25, 31]. Considering that in a country like Sweden, known to have a high housing standard, the existing housing stock displays considerable accessibility problems for the ageing population, this study highlights a large-scale problem for the Western world at large.

Growing evidence show that inaccessible housing is related to dependence in activities of daily living (ADLs) [32] falls [33] and institutionalization [34, 35]. Housing accessibility problems also increase the risk for a lower degree of social participation, which may turn into poorer self-management, isolation and ultimately higher health care needs [36]. Deserving a wider recognition as an important public health issue, improved housing accessibility for senior citizens will likely have positive effects on population health.

It should be noted that the results of our study have wider implications. That is, disability and functional limitations are common also in younger age groups [37], thus knowledge of accessibility problems in ordinary housing has implications for health and quality of life of several sub-groups of the general population.

In line with previous research the results show that entrances, kitchens and bathrooms often have several environmental barriers [38, 39] that generate accessibility problems for people with functional limitations. Kitchens and bathrooms are crucial for many activities of daily living such as being able to cook or shower independently. Thus, these results can be used to develop targeted actions for improving accessibility and independence in ADL and thereby influence health positively. In Sweden, the individual housing adaptation grant is a well-established intervention to eliminate problematic environmental barriers in the home. While this type of intervention is person-centred and target individual needs, our results reveal that the high number of environmental barriers and magnitude of accessibility problems could not possibly be eliminated by housing adaptation grants. Thus, large scale and systematic actions are needed if society wants to support active and healthy ageing for senior citizens [40].

The possibilities to finance large scale upgrading of the existing ordinary housing stock might be questioned, and unfortunately research on cost-effectiveness for housing interventions is rare [41]. A financial evaluation of renovations in multi-dwellings in Sweden showed that, when accomplished simultaneously with pipe replacement, making the bathroom accessible did not cost more than a regular renovation [42]. In fact, it was suggested that effects in terms of postponed or reduced need for home help services, informal care and special housing would make these actions profitable for the municipalities. The result showing how common the environmental barrier *Stairs the only route (no lift/ramp)* at entrances are, underlines the relevance for adopting a societal planning perspective to support active and healthy ageing. Thus, in the most recent governmental commission on housing for the ageing population in Sweden a main recommendation was to provide multi-dwelling estate owners governmental financial support up to 50 % of the cost for the installation of lifts in existing multi-dwellings [7].

To date, the present study is the largest, detailed on-site inventory on environmental barriers and accessibility problems of dwellings in Sweden. To use trained data collectors administering structured assessments at home visits using a research-based instrument has reliability strengths compared to more commonly used self-report inventories [36].

As to representativity, the dwellings included were based on sampling strategies and inclusion criteria in three different original research projects. Since we used functional profiles [27] to estimate the magnitude of accessibility problems for different groups of senior citizens, the weaknesses regarding generalisability were small. Actually, we consider the use of functional profiles to target combinations of functional limitations of senior citizens as a methodological strength. Today, no public statistics are available on the consequences that combinations of different diseases and symptoms generate in terms of functional limitations. The functional profiles used is one way of approaching this complexity and the analyses computed is a way to present a societal perspective on housing accessibility for senior citizens [27]. Though the cases in the simulated analyses could be considered as hypothetical, they still provide an approximation of a reality that is likely applicable for many individuals. Previous studies using authentic cases of older people living in ordinary housing have shown comparable results with regard to housing accessibility problems [33]. However, additional research is needed to further validate the methodology with functional profiles, and a note of caution is therefore in place when interpreting the results. The dwellings located in urban and rural districts in the south of Sweden were fairly representative for the Swedish housing stock regarding building periods as well as housing types.

Due to the complexity of the scoring matrix underlying the accessibility scores generated with the HE, the magnitudes of accessibility problems might be considered challenging to interpret. Optimisation on the scoring matrix and definition of reference values are in progress but further research efforts are needed to present an optimal analysis methodology [43]. Still, with the possibility to make comparisons between different types of dwellings, before and after housing intervention programs, etc. the HE plays an important role. Another limitation is that the HE mainly addresses environmental barriers in relation to physical functional limitations. Assessments on environmental barriers relating to cognitive limitations in a more qualified way than in the present version is needed, as are assessments focusing on supportive features of the built environment.

It should be kept in mind that accessibility is only one out of several aspects of housing shown to be associated with well-being and quality of life. Neighbourhood characteristics and perceived aspects of home and are at least as important for the individual facing disability or age-related functional decline [44, 45]. However, accessibility is an objective aspect relevant for physical planning and housing provision at the societal level.

## Conclusions

Despite high housing standard in the Swedish ordinary housing stock the results show a high prevalence of environmental barriers and substantial accessibility problems for senior citizens with functional limitations. Considering there are many countries with a similar demographic situation and with comparable standard and conditions of the ordinary housing stock, our findings highlight a large-scale problem for the Western world at large. Accessible housing is the basis for active and healthy ageing and a necessity in countries with a strong ageing-in-place policy. To make housing accessible for senior citizens large-scale and systematic efforts involving many actors are required. The results of the present study provide additional impetus to such efforts.

## Abbreviations

ADL, activities of daily living; EA Project, Enable-Age Project; HE, Housing Enabler instrument; PD Project, home and health in people ageing with Parkinson’s disease project; PD, Parkinson’s disease; P-E fit, person-environment fit; Third Age Project, home and health in the third age project

## Appendix

**Table 5:** Categorization of functional limitations/dependence on mobility devices, the Housing Enabler instrument [27]

Functional limitation	Functional limitation category. Items included marked with ‘I’					
	Difficulty interpreting information	Loss of sight	Severe loss of hearing	Limitations in movement	Limitations in upper extremity	Dependence on mobility devices
Difficulty interpreting information	I	-	-	-	-	-
Visual impairment	-	I	-	-	-	-
Blindness	-	I	-	-	-	-
Loss of hearing	-	-	I	-	-	-
Poor balance	-	-	-	I	-	-
Incoordination	-	-	-	I	-	-
Limitations of stamina	-	-	-	I	-	-
Difficulty in moving head	-	-	-	I	-	-
Reduced upper extremity function	-	-	-	-	I	-
Reduced fine motor skills	-	-	-	-	I	-
Loss of upper extremity function	-	-	-	-	I	-
Reduced spine and/or lower extremity function	-	-	-	I	-	-
Dependence on walking aids	-	-	-	-	-	I
Dependence on wheelchair	-	-	-	-	-	I

*Note*: Based on this categorization, four functional profiles were used. Profile I: limitations in movement only; Profile II: limitations in movement and upper extremity; Profile III: limitations in movement, upper extremity and dependence on mobility devices; and Profile IV: limitations in movement, upper extremity, dependence on mobility devices and loss of sight
